# The Neck Exploration of Penetrating Migratory Foreign Body (Crab Leg) Approaching the Right Carotid Sheath: A Rare Case From the United Arab Emirates (UAE)

**DOI:** 10.7759/cureus.74961

**Published:** 2024-12-02

**Authors:** Mutaz M Abualhab, Ali A Kashmoola, Tamer Abo El Ezz, Ragai Gemi

**Affiliations:** 1 Medicine, University of Sharjah College of Medicine, Sharjah, ARE; 2 Otolaryngology-Head and Neck Surgery, Al Qassimi Hospital, Sharjah, ARE

**Keywords:** carotid sheath, crab leg, deep cervical fascia, foreign body impaction, neck exploration

## Abstract

Foreign body ingestion is a problem that commonly presents in almost all otolaryngologic practices. However, less commonly do those foreign bodies perforate, migrate to, and impact the soft tissue of the neck while nearly invading the carotid sheath that accommodates the major neurovascular supply of the head. We report the case of a patient who had radiologic evidence of foreign body impaction and required neck exploration through an external approach to retrieve a crab leg embedded far within the deep cervical fascia.

## Introduction

Foreign body impaction in the throat is a frequently encountered condition in emergency departments, particularly involving fish bones. The direct examination of the fish bone reliably detects the problem and can also help alleviate the patient's anxiety. While plain lateral neck radiography often struggles to detect fish bones because many are not radiopaque, computed tomography (CT) is highly effective at identifying impacted esophageal fish bones with high sensitivity [1]. However, the possibility of fish bone impaction cannot be completely ruled out due to the small chance of false-negative results, especially when symptoms persist [2]. Thus, endoscopic intervention is crucial for patients with ongoing symptoms or a positive CT scan. Currently, there are no well-established algorithms for the diagnosis and management of fish bone impaction. This case involves a patient with a history of swallowing a crab leg, initially presenting with dysphagia and subsequently developing neck pain and swelling on one side. The crab leg was lodged in the soft tissue of the neck, nearly invading the right carotid sheath. The purpose of presenting this case is to raise awareness among clinicians regarding the potential migration of foreign bodies after hypopharyngeal and esophageal penetration and propose a management protocol aimed at optimizing patient outcomes.

## Case presentation

A 40-year-old woman, with a known history of poorly controlled hypertension, arrived at the emergency department due to throat discomfort after accidentally ingesting a crab leg earlier in the day. She reported sharp pain in the lower neck and dysphagia to both solid foods and liquids. The patient denied fever, respiratory discomfort, or drooling. The neck examination revealed tenderness over the right lateral side, exacerbated on extension.

**Figure 1:**
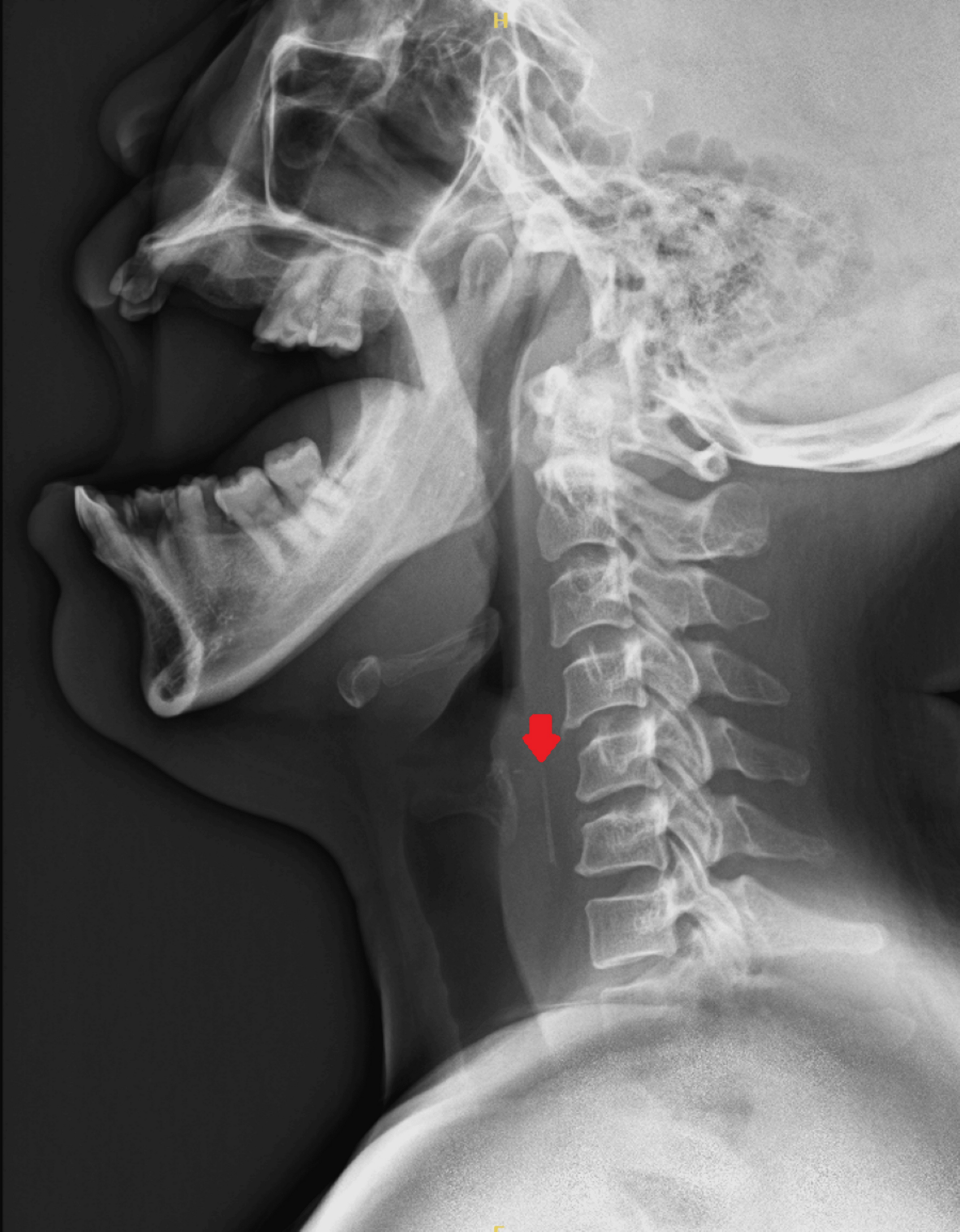
Lateral soft tissue X-ray of the neck revealing suspected foreign body (red arrow).

A flexible nasolaryngoscopy was performed, revealing no direct visualization of the foreign body in the vallecula, base of the tongue, piriform fossa, laryngeal inlet, postcricoid area, or posterolateral wall of the hypopharynx. Additionally, the vocal cords exhibited unrestricted movement. Conservative treatment involving painkillers and antibiotics was initiated, and a lateral neck X-ray did not detect any abnormality initially (Figure 1).

**Figure 2:**
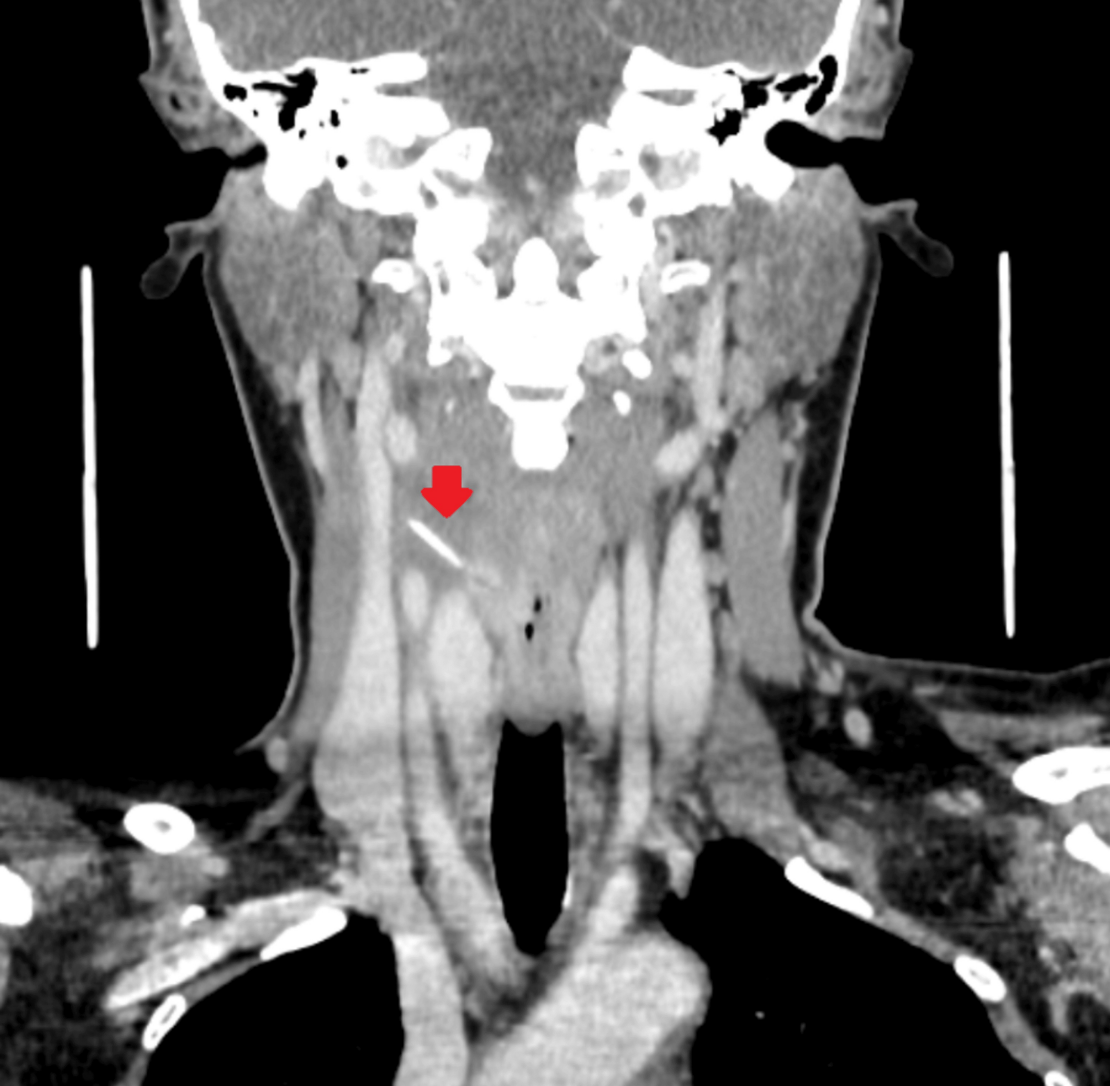
CT scan of the neck showing an impacted foreign body (red arrow) in the soft tissue of the neck. CT: computed tomography

However, five days later, the patient returned to the clinic with persistent throat pain and dysphagia. Esophagoscopy performed under general anesthesia demonstrated the penetration of the lateral esophageal wall, displaying a raised mucosal area with a hole as a crater. Despite this, no direct visualization of the foreign body emerging from it was observed. Consequently, a CT scan of the neck was done that revealed an obliquely oriented prevertebral hyperdense linear foreign body seen opposite the C5/C6 vertebral level, partially embedded in the right esophageal wall with extraluminal extension into the right carotid space, demonstrating mild surrounding soft tissue thickness and hypodensity suggestive of a reactionary process. Notably, the tip of the foreign body was identified 3 mm from the right common carotid artery and 4 mm from the right internal jugular vein (Figure 2). After the CT, we revised the X-ray retrospectively, which revealed a faint foreign body shadow.

**Figure 3:**
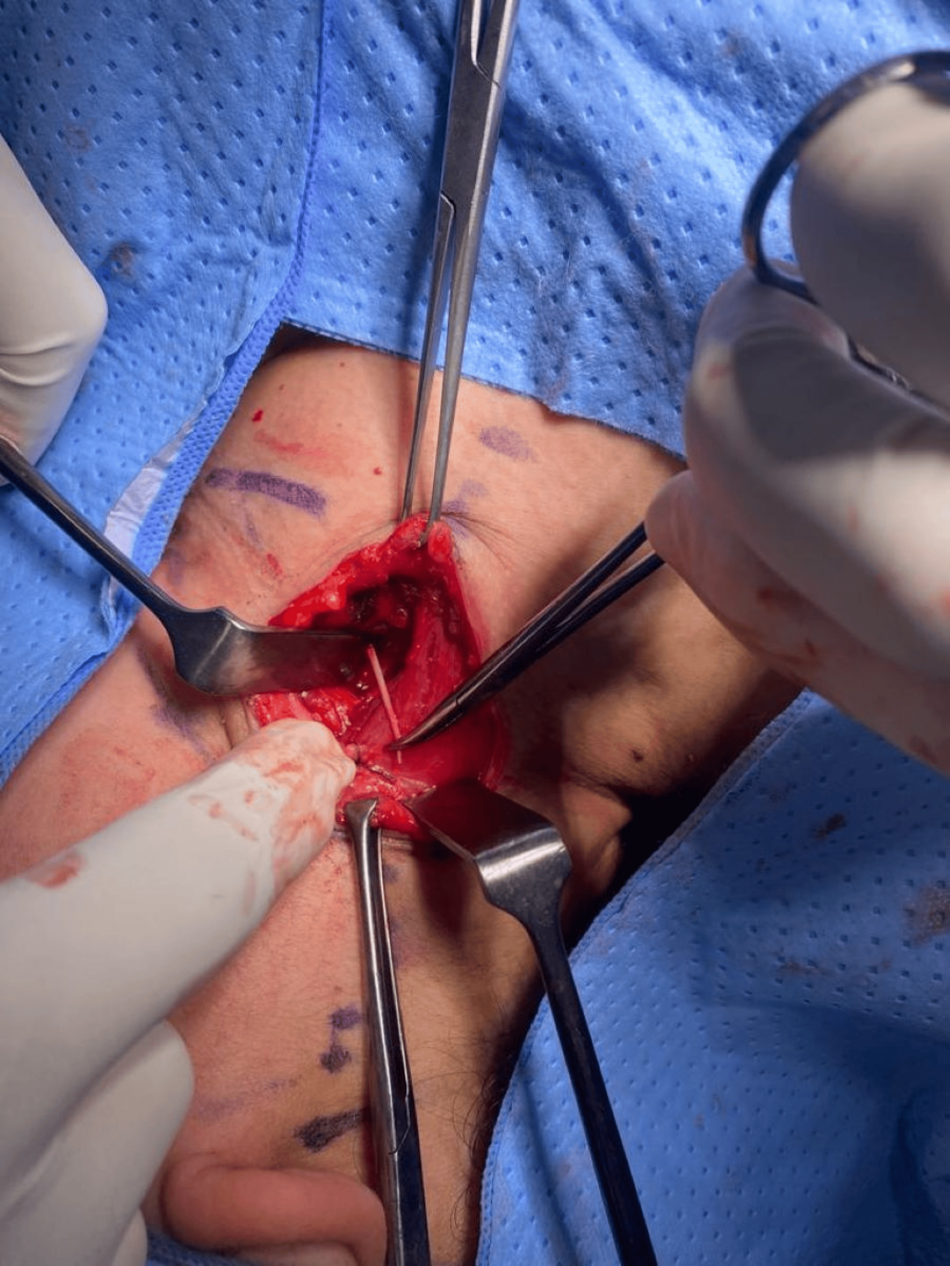
Intraoperative photo demonstrating impacted crab leg.

**Figure 4:**
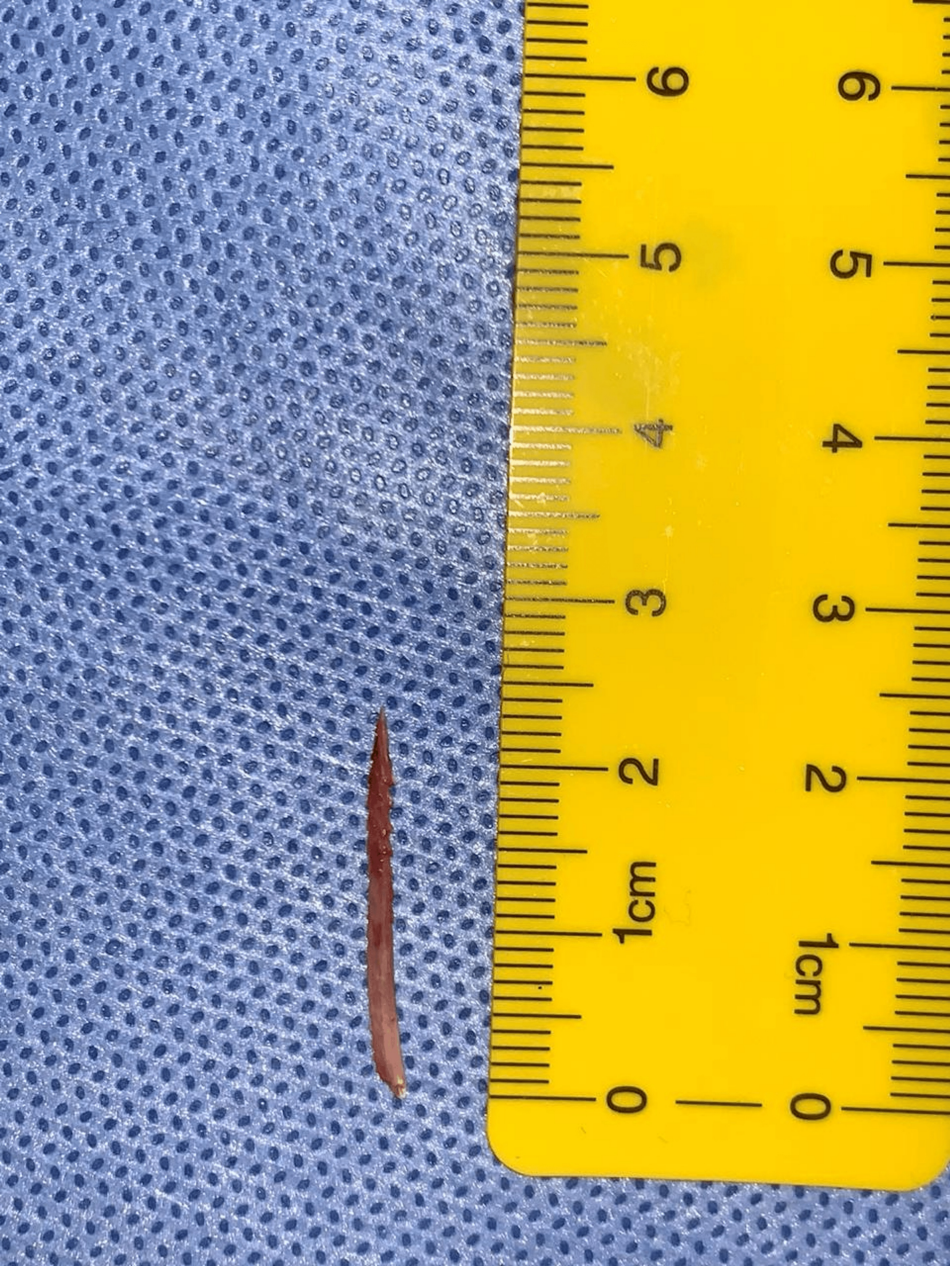
Crab leg extracted from the soft tissue of the neck.

As a result of these findings, the patient was admitted for a potential neck exploration. In the first few days of admission, the patient was doing well on intravenous antibiotics, showing no signs of fever and reporting reduced pain compared to before. Additionally, she also admitted to having a dry cough with mild expectoration of blood. On day 3, after getting the informed verbal and written consent, she was kept nil per os for neck exploration under general anesthesia. In the operation theatre, the neck area was marked, and appropriate cleaning and draping were done. An incision was made extending from the anterior head of the sternocleidomastoid muscle to the thyroid ala (Figure 3). The skin was separated, the platysma was dissected, and the sternocleidomastoid was retracted (the carotid sheath was retracted laterally). The foreign body was palpated before removing it successfully (Figure 4), and no pus collection was seen. The closure of the wound was done in layers, and the skin was sutured using silk with the insertion of a size 12 vacuum drain. Postoperatively, the patient did not have any complications, and the drain yielded 10 mL of serosanguinous fluid. The patient was started on intravenous antibiotics and was kept fasting for 12 hours and then transitioned to a soft diet for the first 24 hours, followed by a regular diet before she was discharged on postoperative day 2.

## Discussion

Foreign body ingestion is a relatively common issue, increasing in incidence over the past two decades [3]. The most common locations for ingested foreign body impaction include the upper esophagus, followed by the pharyngoesophageal junction, transjunctional, hypopharynx, and oropharynx [4]. Rarely do foreign bodies penetrate the wall of the digestive tract, and even more rarely do they migrate into the soft tissue and viscera of the neck. The risk of penetration is strongly influenced by the sharpness and orientation of the foreign body. Sharper and horizontally oriented objects are more likely to penetrate the mucosal wall. Higher rates of penetration occur in the hypopharynx and cervical esophagus, facilitated by the strong contractions of the cricopharyngeal and hypopharyngeal muscles as they propel the food bolus into the esophagus. The perforation and migration of the foreign body can introduce bacteria and act as a nidus for recurrent refractory infections, resulting in complications such as parapharyngeal and retropharyngeal abscesses. The spread of the infection toward the mediastinum can lead to life-threatening mediastinitis. The foreign body may also travel to the thyroid gland, as previously described in a case reported in the United Arab Emirates (UAE) [5].

**Figure 5:**
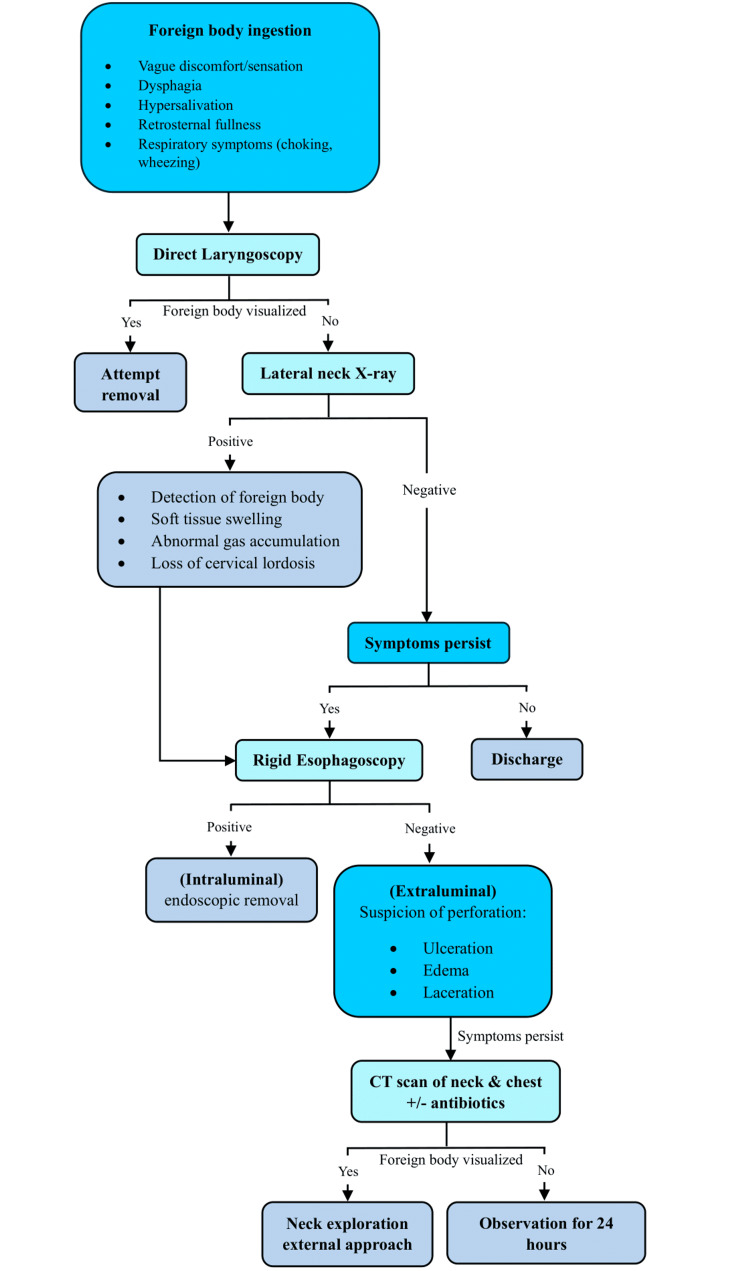
Management and treatment algorithm for suspected foreign body ingestion and penetration. CT: computed tomography

Vascular complications have also been discussed in the literature with aortoesophageal fistula being the most common, followed by innominate esophageal fistula, carotid artery injuries including thrombosis, and rupture [6]. In this case, the crab leg was very close to the carotid artery but did not puncture it. Grim consequences would have been expected as the patient had very high blood pressure. As for any patient presenting with a history suggestive of foreign body ingestion, a lateral neck X-ray is one of the most important preliminary tools to provide diagnostic evidence. Certain findings that indicate pathological films include 1) the detection of the foreign body, 2) soft tissue swelling, 3) abnormal gas accumulation, and 4) the loss of cervical lordosis [7]. According to one study, the presence of abnormal radiopaque density and air lucency was identified as the two signs with the highest diagnostic value, accurately diagnosing 84.3% and 66.8% of patients with esophageal foreign bodies, respectively [8]. In spite of that, lateral neck X-rays fail to detect whether migration has occurred or not. Thus, when there is a high suspicion of foreign body impaction and patients continue to have symptoms, rigid esophagoscopy is typically indicated. This patient had a negative esophagoscopy, evidenced by the presence of a raised mucosa with a hole suggesting the extraluminal penetration of the foreign body across the esophageal wall. Even then, maneuvering the endoscope probe around the area revealed no foreign body. Despite this, the patient continued to have symptoms, prompting a CT scan to be ordered, which localized the foreign body within the soft tissue of the neck, in close proximity to the carotid sheath. Consequently, a neck exploration approach was pursued (Figure 5).

## Conclusions

In conclusion, to increase awareness among emergency and ENT physicians, we propose a management algorithm for patients presenting with a history suggestive of swallowing sharp foreign bodies such as fish bones, seafood, or pins. Foreign body ingestion and penetration may lead to perforation and even life-threatening complications necessitating attention to avoid further complications and improve patient outcomes.
